# *Egr2* Deletion in Autoimmune-Prone C57BL6/*lpr* Mice Suppresses the Expression of Methylation-Sensitive *Dlk1*-*Dio3* Cluster MicroRNAs

**DOI:** 10.4049/immunohorizons.2300111

**Published:** 2023-12-28

**Authors:** Zhuang Wang, Bettina Heid, Jianlin He, Hehuang Xie, Christopher M. Reilly, Rujuan Dai, S. Ansar Ahmed

**Affiliations:** * Department of Biomedical Sciences and Pathobiology, Virginia-Maryland College of Veterinary Medicine, Virginia Tech, Blacksburg, VA; † Epigenomics and Computational Biology Lab, Fralin Life Sciences Institute at Virginia Tech, Blacksburg, VA; ‡ Department of Cell Biology and Physiology, Edward Via College of Osteopathic Medicine, Blacksburg, VA

## Abstract

We previously demonstrated that the upregulation of microRNAs (miRNAs) at the genomic imprinted *Dlk1*-*Dio3* locus in murine lupus is correlated with global DNA hypomethylation. We now report that the *Dlk1-Dio3* genomic region in CD4^+^ T cells of MRL/*lpr* mice is hypomethylated, linking it to increased *Dlk1-Dio3* miRNA expression. We evaluated the gene expression of methylating enzymes, DNA methyltransferases (DNMTs), and demethylating ten-eleven translocation proteins (TETs) to elucidate the molecular basis of DNA hypomethylation in lupus CD4^+^ T cells. There was a significantly elevated expression of *Dnmt1* and* Dnmt3b,* as well as *Tet1* and *Tet2*, in CD4^+^ T cells of three different lupus-prone mouse strains compared to controls. These findings suggest that the hypomethylation of murine lupus CD4^+^ T cells is likely attributed to a TET-mediated active demethylation pathway. Moreover, we found that deletion of early growth response 2 (*Egr2*), a transcription factor gene in B6/*lpr* mice markedly reduced maternally expressed miRNA genes but not paternally expressed protein-coding genes at the *Dlk1*-*Dio3* locus in CD4^+^ T cells. EGR2 has been shown to induce DNA demethylation by recruiting TETs. Surprisingly, we found that deleting *Egr2* in B6/*lpr* mice induced more hypomethylated differentially methylated regions at either the whole-genome level or the *Dlk1*-*Dio3* locus in CD4^+^ T cells. Although the role of methylation in EGR2-mediated regulation of *Dlk1-Dio3* miRNAs is not readily apparent, these are the first data to show that in lupus, *Egr2* regulates *Dlk1-Dio3* miRNAs, which target major signaling pathways in autoimmunity. These data provide a new perspective on the role of upregulated EGR2 in lupus pathogenesis.

## Introduction

Systemic lupus erythematosus (SLE) is a prototypical chronic autoimmune disease characterized by autoimmune attacks on multiple organs. Due to the complexity of this disease, the precise etiopathogenesis of SLE remains elusive. The onset and progress of SLE are attributed to the interaction of multiple factors, including genetics, epigenetics, hormones, and environmental factors. Recent studies have increasingly acknowledged the interplay of two major epigenetic factors, DNA methylation and microRNAs (miRNAs), in autoimmune diseases (1–3).

DNA methylation is an epigenetic process that regulates gene expression at the transcriptional level via affecting DNA accessibility at the regulatory regions (4). The importance of hypomethylation of CD4^+^ T cells in lupus pathogenesis was strikingly demonstrated by the findings that adoptive transfer of demethylated normal human or murine CD4^+^ T cells into nonautoimmune syngeneic recipient mice induced lupus-like disease (5–7). The whole-genome bisulfite sequencing analysis revealed a global DNA methylation loss. There was a common hypomethylation of type I IFN signature genes in CD4^+^ T cells, CD19^+^ B cells, CD14^+^ monocytes, and neutrophils, resulting in heightened type I IFN signaling in human patients with lupus (8–12). In addition to type I IFN signature genes, many other hypomethylated genes (such as *CD70*, *ITGAL*, *IL10*, and *IL17*) have been identified in lupus cells, which contributed to abnormal immune cell activation and inflammation in lupus (13). miRNAs are small non–protein-coding RNAs that regulate gene expression mainly at the post-transcriptional level through complementary binding to target mRNAs (14–16). In both human and murine lupus, a number of dysregulated miRNAs have been identified and linked to the disease pathogenesis (17, 18). Of these dysregulated miRNAs in lupus, a group of miRNAs is located at the genomic imprinted delta-like homolog 1–type 3 iodothyronine deiodinase (*Dlk1*-*Dio3*) locus, which possesses the largest miRNA cluster in human and murine genome (3, 19). In addition, the upregulation of *Dlk1*-*Dio3* miRNAs has also been implicated the pathogenesis of multiple sclerosis (MS) and experimental autoimmune encephalomyelitis (a rodent model of MS) (20, 21).

The interplay between DNA methylation and miRNAs has been documented in autoimmune lupus. The upregulated miR-21, miR-148a, and miR-126 in lupus contribute to DNA hypomethylation by repressing DNMTs directly or indirectly (22, 23). In contrast, the upregulation of X-chromosome–linked miRNAs in women patients with lupus is likely the result of the loss of methylation at the X-chromosome (24). We have also reported the association between the upregulation of *Dlk1*-*Dio3* miRNAs and global DNA hypomethylation in the lymphocytes of MRL/*lpr* mice (19). DNA demethylation treatment of splenic T cells of control MRL mice significantly increased the expression of *Dlk1*-*Dio3* miRNAs, suggesting an important role of DNA methylation in regulation of *Dlk1*-*Dio3* miRNAs expression (19). However, the molecular mechanism underlying the DNA hypomethylation and the upregulation of *Dlk1*-*Dio3* miRNAs in lupus CD4^+^ T cells remains unclear.

Early growth response 2 (EGR2) protein is a member of the EGR zinc finger transcription factor family. The critical roles of EGR2 in regulating T cell development and activation, adaptive immune response, inflammatory cytokine production, and autoimmunity have been widely investigated recently and well-acknowledged (25–32). In contrast to the finding that conditional *Egr2* gene deletion in normal C57BL/6 (B6) mice led to the production of anti-nuclear autoantibodies and increased total Ig G (IgG) production mice (28), conditional *Egr2* gene deletion in autoimmune prone B6/*lpr* mice significantly reduced anti-dsDNA autoantibodies and total Igs production (31). This suggests that the immune regulatory role of EGR2 is complex and depends on the immune status and the reasons why *Egr2* deletion had different immune changes in B6 and B6/*lpr* need to be further investigated.

This study aims to understand the molecular mechanism underlying the DNA hypomethylation and the upregulation of *Dlk1*-*Dio3* miRNAs in lupus CD4^+^ T cells. We found that the ten-eleven translocation protein (TET)–mediated active DNA demethylation pathway, rather than the DNA methyltransferase (DNMT)–mediated passive DNA demethylation pathway, is more likely to contribute to the DNA hypomethylation of lupus CD4^+^ T cells. Further, we report that *Egr2* deletion in B6/*lpr* mice significantly reduced *Dlk1*-*Dio3* miRNAs expression in CD4^+^ T cells. Surprisingly, *Egr2* deletion led to a global DNA methylation loss and hypomethylation at *Dlk1*-*Dio3* locus in CD4^+^ T cells. To our knowledge, our findings are the first to show that EGR2 regulates the expression of miRNAs at the genomic imprinted *Dlk1-Dio3* genomic locus, although the mechanisms need further investigation.

## Materials and Methods

### Mice

All experimental animal procedures and housing have been approved by the Institutional Animal Care and Use Committee of Virginia Polytechnic Institute and State University. In this study, MRL (MRL/MpJ) and autoimmune-prone MRL/*lpr* (MRL/MpJ-Fas^lpr^/J), B6/*lpr* (B6.MRL-Fas^lpr^/J), and B6.*sle123* (B6.NZMSle1/Sle2/Sle3) breeders were purchased from The Jackson Laboratory and bred in-house. We also purchased normal B6 (C57BL/6J) breeders from The Jackson Laboratory. The conditional CD2-specific *Egr2^−/−^*B6/*lpr* strain was developed in our laboratory and described in detail in a recent publication (31). The *Egr2^−/−^*B6/*lpr* and littermate *Egr2*^fl/fl^B6/*lpr* mice were used for experiments as knockout and control groups, respectively. The sexes and ages of the animals used for the experiments are specified in the figure legends.

All mice were maintained in our Association for Assessment and Accreditation of Laboratory Animal Care–certified animal facility at the Virginia-Maryland College of Veterinary Medicine, Virginia Tech. The mice were fed with the commercial 7013 NIH-31 modified 6% mouse/rat sterilizable diet (Harlan Laboratory, Madison, WI) and given water ad libitum. We euthanized mice by CO_2_ asphyxiation followed by cervical dislocation or heart puncture at the designated age according to the Institutional Animal Care and Use Committee–approved protocol.

### Splenic lymphocyte preparation and CD4^+^ cell isolation

Whole splenic lymphocytes were prepared following standard laboratory protocols described in detail previously (33, 34). Per the manufacturer’s protocol, splenic CD4^+^ T cells were isolated from whole splenic lymphocytes with anti-CD4 (L3T4) MicroBeads (Miltenyi Biotec, San Diego, CA).

### RNA extraction

As we previously reported (19, 34), total RNA containing small RNA was extracted from CD4^+^ T cells with an miRNeasy mini kit (Qiagen) by following the manufacturer’s procedures. On-column DNA digestion was conducted to remove residual DNA in the RNA samples. The concentration and purity of RNA were assessed by NANODROP 2000 spectrophotometer (Thermo Fisher Scientific). Samples with OD_260/280_ ratios of around 2.0 were used for TaqMan miRNA assay and quantitative RT-PCR (RT-qPCR) assays.

### Reverse transcription and RT-qPCR

The SuperScript IV First-Strand Synthesis System (Invitrogen, Waltham, MA) was used to synthesize the first-strand cDNA from RNA samples per the manufacturer’s protocol. RT-qPCR was conducted using Power SYBR green PCR mix (Applied Biosystem, Waltham, MA) per our previous report (34). The RT-qPCR primers for *Dnmt1/3a/3b* and *Tet1/2/3* genes were designed with the Primer quest tool and synthesized by Integrated DNA Technologies (IDT, San Diego, CA). All the primer sequences are listed in Table I.

**Table I.** Primer sequences

Gene Name	5′ to 3′	Sequence
Tet1	Forward	GAGCCTGTTCCTCGATGTGG
	Reverse	CAAACCCACCTGAGGCTGTT
Tet2	Forward	AACCTGGCTACTGTCATTGCTCCA
	Reverse	ATGTTCTGCTGGTCTCTGTGGGAA
Tet3	Forward	TCCGGATTGAGAAGGTCATC
	Reverse	CCAGGCCAGGATCAAGATAA
DNMT1	Forward	CCTAGTTCCGTGGCTACGAGGAGAA
	Reverse	TCTCTCTCCTCTGCAGCCGACTCA
DNMT3a	Forward	GCCGAATTGTGTCTTGGTGGATGACA
	Reverse	CCTGGTGGAATGCACTGCAGAAGGA
DNMT3b	Forward	TCAGTGACCAGTCCTCAGACACGAA
	Reverse	TCAGAAGGCTGGAGACCTCCCTCTT

The *Dlk1* (Assay ID Mm00494478_m1) and *Dio3* (Assay ID Mm00548953_s1) Taqman assays were purchased from Thermo Fisher Scientific to evaluate the expression of *Dlk1* and *Dio3* gene expression by following the manufacturer’s instruction. Strain-specific RT-qPCR was performed to evaluate the expression of *Rtl1* and *antisense-Rtl1* (*asRtl1*) genes by following a previously published protocol (35). The RT-qPCRs were performed with the 7500 Fast Real-Time PCR system (Applied Biosystem). The relative expression level of mRNAs was normalized to *18s* and calculated by the 2^−ΔΔCt^ method.

### TaqMan miRNA assay

The TaqMan miRNA assays (Applied Biosystems) was used to quantify the expression level of miRNAs (miR-154, miR-127, miR-379, miR-433, miR-300, miR-21, miR-503, miR-182, and miR-183) as we previously reported (19, 36). The relative expression level of miRNAs was normalized to endogenous control snoRNA202 and calculated by the 2^−ΔΔCt^ method.

### Genome-wide bisulfite sequencing and data analysis

CD4^+^ T cell DNA was isolated with DNeasy blood and tissue kit (Qiagen). The MRL and MRL/*lpr* CD4^+^ T cell DNA samples (*n* = 3 each strain) were sent to Zymo Research for Methyl-MiniSeq library preparation and sequencing. Briefly, 300 ng of genomic DNA was sequentially digested with 60 U of TaqαI and 30 U of MspI (NEB) and then purified by DNA Clean & Concentrator-5 (Zymo Research). The purified DNA fragments were ligated to preannealed adapters containing 5′-methyl-cytosine according to Illumina’s specified guidelines. Adaptor-ligated fragments of 150–250 bp and 250–350 bp in size were recovered from a 2.5% NuSieve 1:1 agarose gel using Zymoclean gel DNA recovery kit and then bisulfite-treated using the EZ DNA Methylation-Lightning kit. Amplification was performed, and the resulting products were purified for sequencing on an Illumina HiSeq. The *Egr2^−/−^*B6/*lpr* and *Egr2^fl/fl^*B6/*lpr* CD4^+^ T cell DNA samples (*n* = 2 each) were sent to CD Genomics (Shirley, NY) for classical reduced representative bisulfite sequencing (RRBS) library construction and sequencing. Briefly, the genomic DNA was digested by Mspl (NEB) followed by blunt-end repaired, A-tailed and adaptor ligation. Size selection was performed using AMPure XP beads (Beckman Coulter) to obtain DNA fractions enriched for the most CpG-rich regions in the range of 150–300 bp. Subsequently, bisulfite conversion and library preparation were conducted using the ZYMO EZ DNA Methylation-Gold kit following the manufacturer’s instructions. Library quality and quantity were assessed with Qubit 2.0 DNA HS assay and TapeStation high-sensitivity D1000 assay. Equimolar pooling of libraries was performed based on quality control values and sequenced on an Illumina PE150.

The raw sequencing data were processed and analyzed as described following. Briefly, the adapters and low-quality reads were trimmed by Trim Galore (version 0.6.7). After trimming the adapters and filtering low-quality reads, sequencing reads were mapped to mouse GRCm39 genome assembly using Bismark with Bowtie2 (version V0.23.1) (37). To determine the differentially methylated sites (DMSs) (*Egr2^−/−^*B6/*lpr* versus control *Egr2^fl/fl^*B6/*lpr*; MRL/*lpr *versus control MRL), we performed the Benjamini–Hochberg procedure to adjust the *p* value, and the CpG sites with adjusted *p* value less than 0.05 were determined as DMSs by R (version 4.2.1). The DMSs adjacent to each other (less than 500 bp in the distance) were merged as candidates for DMRs. To ensure the methylation changes are consistent throughout the entire region, we further selected DMRs with more than 80% of DMSs showing 0.1 or more methylation difference, and the overall methylation difference is higher than 0.1.

### Statistical analysis

Th equantitative data are presented in graphs as means ± SD. Student *t* tests were analyzed by GraphPad Prism software to determine the statistical significance between two treatment groups (MRL/*lpr versus* MRL; B6*/lpr versus* B6, B6.*sle123 versus* B6; *Egr2*^−/−^B6/*lpr versus Egr2*^fl/fl^B6/*lpr*). *p < 0.05, **p < 0.01, ***p < 0.001, ****p < 0.0001.

## Results

### *Dlk1-Dio3* locus is hypomethylated in MRL/*lpr* CD4^+^ T cells

**FIGURE 1.**
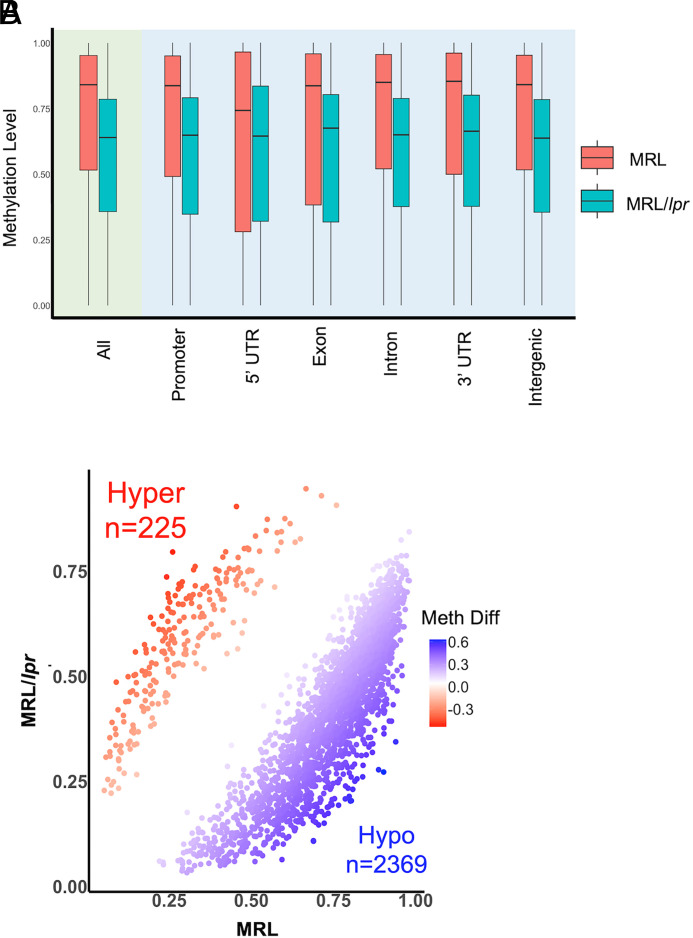
DNA hypomethylation in MRL/*lpr* CD4^+^ T cells when compared with control MRL CD4^+^ T cells. RRBS assays were performed to profile the DNA methylation in purified splenic CD4^+^ T cells from 14–15-wk-old female MRL/*lpr* and sex- and age-matched control MRL mice (*n* = 3 each). DMSs and DMRs were identified as we described in detail in “Materials and Methods.” (**A**) Mean methylation level of DMSs at different genomic regions showed hypomethylation (Hypo) in CD4^+^ T cells of MRL/*lpr* mice. (**B**) Number of hypermethylated (Hyper) DMRs (false discovery rate [FDR] < 0.05) and hypomethylated DMRs (FDR < 0.05) in CD4^+^ T cells of MRL/*lpr* compared with MRL mice. Meth Diff, methylation difference; UTR, untranslated region.

Our previous study has shown the correlation between global DNA hypomethylation and upregulation of *Dlk1*-*Dio3* miRNAs in MRL/*lpr* CD4^+^ T cells (19). DNA methylation plays a major role in the regulation of the expression of genes located at the genomic imprinted *Dlk1*-*Dio3* locus (19, 38, 39). Since the *Dlk1*-*Dio3* region is too large for the target bisulfite sequencing, we performed genome-wide methylation analysis with double enzyme RRBS to determine whether the *Dlk1*-*Dio3* locus is differentially methylated between the CD4^+^ T cells from female MRL/*lpr* and MRL mice. We obtained around 32 million uniquely aligned reads, which covered 54–59% of cytosines in the CpG context for MRL/*lpr* and MRL mice. Consistently, we observed a global DNA hypomethylation in MRL/*lpr* CD4^+^ T cells and a methylation loss at different functional genomic regions (Fig. 1A). Further, there were more hypomethylated (*n* = 2369) differentially methylated regions (DMRs) than hypermethylated (*n* = 225) DMRs in MRL/*lpr* CD4^+^ T cells when compared with control MRL CD4^+^ T cells (Fig. 1B). Importantly, we identified three DMRs located at the *Dlk1*-*Dio3* region, and all three of these DMRs were hypomethylated (Table II). These data demonstrated an association between DNA demethylation at the *Dlk1*-*Dio3* locus and the upregulation of *Dlk1*-*Dio3* miRNAs in MRL/*lpr* CD4^+^ T cells.

**Table II.** DMRs at *Dlk1-Dio3* region in MRL/*lpr* compared with MRL mice

Chrom	Start	End	Length	nCG	Meth Diff	MRL/*lpr* Mean Meth	MRL Mean Meth
chr12	109459844	109459901	58	7	−0.20677	0.19150	0.39827
chr12	109472727	109472844	118	9	−0.27901	0.58034	0.85936
chr12	109825628	109825805	178	4	−0.298149	0.62189	0.92004

Chrom, chromosome; Meth Diff, methylation difference; nCG, number of cytosine-guanine.

### Increased *Dnmts* and *Tets* gene expression in MRL/*lpr* CD4^+^ T cells

**FIGURE 2.**
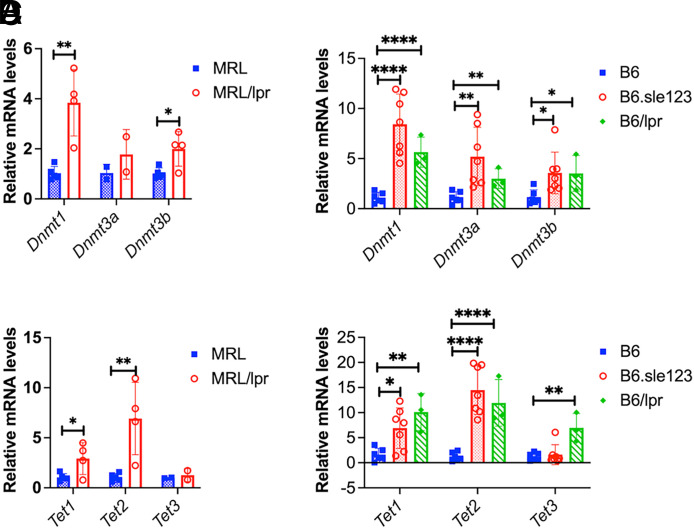
Increased *Dnmts* and *Tets* gene expression in CD4^+^ T cells of lupus-prone mice. RT-qPCR was performed to quantify the expression levels of *Dnmt* and *Tet* genes in purified splenic CD4^+^ T cells from female MRL/*lpr* (14–15 wk old), B6.*sle123* (5–6 mo old), and B6/*lpr* (5**-**6 mo old) mice and their respective sex- and age-matched control MRL and B6 mice. (**A** and **C**) Summary graphs show the significant upregulation of *Dnmt1/3b* (A) and *Tet1/2* (C) genes in CD4^+^ T cells of MRL/*lpr* mice compared with MRL controls (*n* ≥ 2 each). (**B** and **D**) Summary graphs show a significant increase of *Dnmt1/3a/3b* (B) and *Tet1/2/3* (D) in two other types of lupus-prone mice, B6/*lpr* and B6.*sle123* mice compared with control B6 mice (*n* ≥ 3 each). Horizontal bars in the summary graphs indicated means ± SD. Unpaired Student *t* test was performed to determine the statistical significance in the comparison of gene expression levels between two groups (MRL *vs.* MRL/*lpr*, B6.*sle123 versus* B6, and B6/*lpr versus* B6). Statistical significance is indicated by asterisks: *p < 0.05, **p < 0.01, ****p < 0.0001.

Reduced expression/activity of DNMT1 and/or DNMT3a/3b has been identified in the PBMCs or CD4^+^ T cells of human patients with lupus, which contributed to the DNA hypomethylation in human lupus cells (40–42). To our surprise, we found a significant increase in the expression of *Dnmt1* and *Dnmt3b* genes, whereas the expression of *Dnmt3a* gene was unchanged in MRL/*lpr* CD4^+^ T cells when compared with MRL CD4^+^ T cells (Fig. 2A). The increase of *Dnmt* gene expression was also observed in the CD4^+^ T cells of other lupus-prone mice models, B6/*lpr* and B6.*sle123* (Fig. 2B). This suggests that reduced DNA methylation in the murine lupus CD4^+^ T cells is likely not due to the reduced expression of *Dnmt* genes. Current studies have implied that the TETs mediated the active demethylation pathway in the DNA hypomethylation in human lupus (13, 43, 44). Notably, we found that *Tet1* and *Tet2* expression was significantly increased in MRL/*lpr* CD4^+^ T cells when compared with MRL CD4^+^ T cells (Fig. 2C). There was no significant change in the expression of *Tet3* gene. Further, we demonstrated the upregulation of *Tet1*, *Tet2*, and *Tet3* genes in CD4^+^ T cells of other two lupus strains, B6/*lpr* and B6.*sle123* (Fig. 2D). These data strongly suggested that the TET-mediated active demethylation pathway likely plays a major role in driving DNA demethylation in murine lupus CD4^+^ T cells.

### Conditional deletion of *Egr2* gene specifically inhibits the expression of maternally expressed *Dlk1-Dio3* miRNAs in B6/*lpr* mice

**FIGURE 3.**
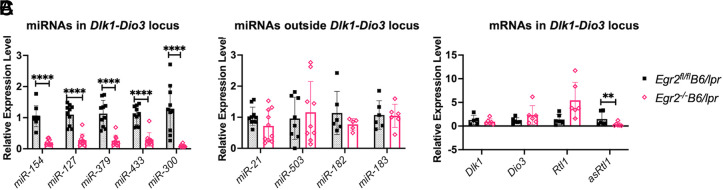
*Egr2* deletion in B6/*lpr* mice significantly suppresses maternally expressed *Dlk1-Dio3* miRNAs and mRNA expression. The expression levels of miRNAs and mRNAs in splenic CD4^+^ T cells from *Egr2*^−/−^B6/*lpr* at 5–6-mo-old and littermate control *Egr2*^fl/fl^B6/*lpr* mice were quantified by TaqMan miRNA assays and RT-qPCR. Both female and male mice were used. (**A**) Summary graph shows a significant decrease in the expression of miRNAs located at *Dlk1-Dio3* locus in *Egr2*^−/−^B6/*lpr* compared with controls. (**B**) Summary graph shows that *Egr2* deletion had no obvious effect on the expression of miR-21, miR-503, miR-182, and miR-183, which are located outside the *Dlk1-Dio3* locus. Two independent experiments were performed with four and six mice for each experiment. (**C**) Graph shows that *Egr2* deletion in B6/*lpr* mice significantly inhibited the expression of maternally expressed non–protein coding gene *asRtl1* but not paternally expressed protein-coding genes, including *Dlk1*, *Dio3*, and* Rtl1.* Horizontal bars in the summary graphs indicate the means ± SD (*n* ≥ 6). Unpaired Student *t* test was performed to determine the statistical significance in the comparison of gene expression levels between *Egr2*^fl/fl^B6/*lpr* and *Egr2*^−/−^B6/*lpr*. **p < 0.01, ****p < 0.0001.

DNA methylation is tightly regulated in a cell-specific manner during immune cell development and differentiation (45). Specific transcription factors have been shown to be required for regulating site-specific DNA methylation gene expression activities by recruiting DNMTs or TETs to their binding sites (46). Of particular interest, in a recent study, EGR2 was found to play a major role in the turnover of DNA methylation during the differentiation of human monocytes by recruiting TET2 to its binding sites (47). This study suggested an important epigenetic regulatory role of EGR2 in immune cells, an aspect not well known yet. We have recently developed conditional *Egr2^−/−^*B6/*lpr* mice to investigate the autoimmune regulatory role of EGR2 in the autoimmune lupus context. To test the potential epigenetic regulatory role of EGR2 in lupus CD4^+^ T cells, we evaluated the expression of selected methylation-sensitive *Dlk1*-*Dio3* miRNAs, which are highly upregulated in lupus (e.g., miR-127, miR-154, miR-300, miR-279, and miR-433). Excitingly, we found that the expression of these *Dlk1*-*Dio3* miRNAs was significantly reduced in CD4^+^ T cells of *Egr2^−/−^*B6/*lpr* mice when compared with the cells from control *Egr2^fl/fl^*B6/*lpr* mice (Fig. 3A). Notably, there were no obvious differences observed in miRNAs outside the *Dlk1*-*Dio3* locus (e.g., miR-21, miR-182, miR-183, and miR-503) (Fig. 3B). In addition to the maternally expressed non–protein-coding miRNA genes, the *Dlk1*-*Dio3* region also contains paternal protein-coding genes, such as *Dlk1*, *Rtl1*, and *Dio3* (3). To distinguish *asRtl1* and *Rtl1* transcript, we performed strain-specific RT-qPCR as previously reported (35). Although *Egr2* deletion significantly suppressed maternally expressed *asRtl1*, which encodes multiple miRNAs, *Egr2* deletion had no obvious effect on the expression of paternally expressed *Dlk1*, *Rtl1*, and *Dio3* genes (Fig. 3C). Together, these data demonstrated that EGR2 specifically regulates the expression of maternally expressed miRNA genes but not paternally expressed protein-coding genes at the genomic imprinted *Dlk1*-*Dio3* locus in B6/*lpr* mice.

### Conditional *Egr2* deletion leads to methylation change at *Dlk1-Dio3* locus in B6/*lpr* mice

**FIGURE 4.**
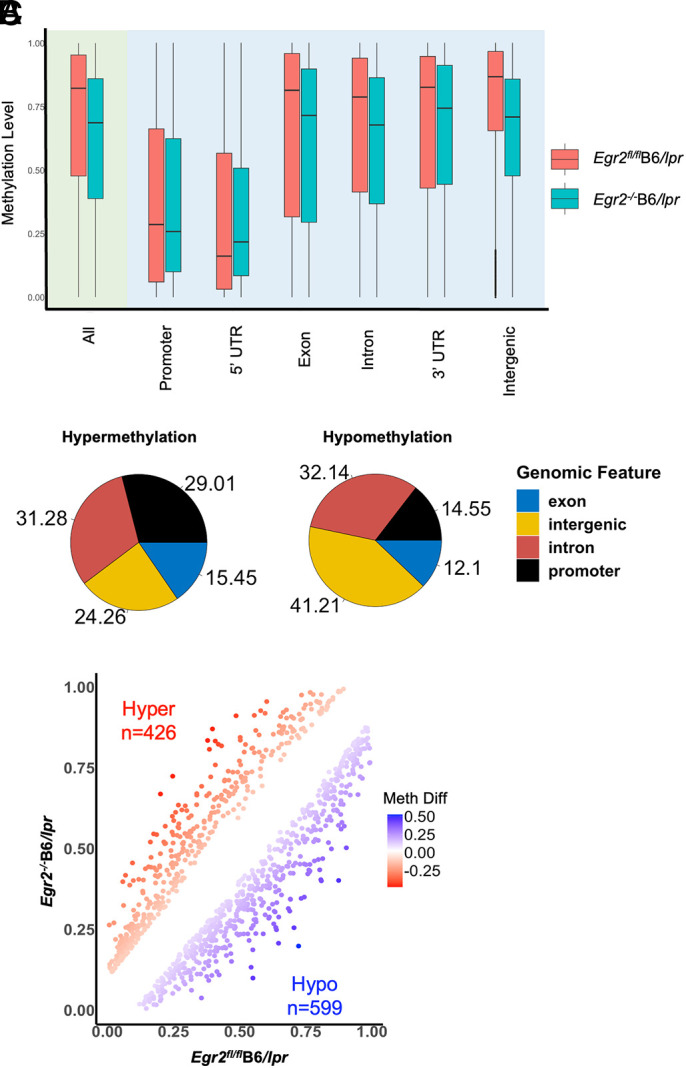
*Egr2* deletion in B6/*lpr* mice induces a methylation loss at a genome-wide level. RRBS assays were performed to profile the DNA methylation in purified splenic CD4^+^ T cells from female *Egr2*^−/−^B6/*lpr* mice at ∼5 mo old and sex-matched littermate control *Egr2*^fl/fl^B6/*lpr* mice (*n* = 2 each). DMSs and DMRs were identified as we described in detail in “Materials and Methods.” (**A**) Mean methylation levels of DMSs at different genomic regions showed reduced methylation at a whole-genome level and at different genomic regions except the 5′-untranslated region (UTR) in CD4^+^ T cells of *Egr2*^−/−^B6/*lpr* mice compare with control *Egr2*^fl/fl^B6/*lpr* mice. (**B**) Pie charts showed the distribution of hypermethylated (Hyper) DMSs and hypomethylated (Hypo) DMSs at the four genomic regions. (**C**) Graph showed the number of hypermethylated DMRs (false discovery rate [FDR] < 0.05) and hypomethylated DMRs (FDR < 0.05) in CD4^+^ T cells of *Egr2*^−/−^B6/*lpr* compared with control *Egr2^fl/fl^*B6/*lpr* mice. Meth Diff, methylation difference.

DNA methylation plays an essential role in the expression of genomic imprinted genes. The above data indicated that *Egr2* deletion suppressed methylation-sensitive *Dlk1*-*Dio3* miRNAs (Fig. 3). With the newly emerged epigenetic regulatory role of EGR2, we therefore investigated whether *Egr2* deletion promotes the methylation at *Dlk1*-*Dio3* locus to suppress *Dlk1*-*Dio3* miRNA expression. We performed RRBS to measure *Egr2* deletion–induced DNA methylation changes in CD4^+^ T cells of B6/*lpr* mice. We obtained around 36 million uniquely aligned reads per sample, which covered 62.5 and 65% of cytosines in the CpG context for *Egr2*^−/−^B6/*lpr* and *Egr2*^fl/fl^B6/*lpr*, respectively. To our surprise, we observed that *Egr2* deletion led to a genome-wide methylation loss (Fig. 4A). We noticed the difference in the distribution of *Egr2* deletion–induced hypermethylated DMSs and hypomethylated DMSs in the different functional genomic regions (Fig. 4B). There were 29.01% of hypermethylated DMSs located at the promoter region, whereas only 14.55% of the hypomethylated DMSs were distributed at the promoter region. There was a higher percentage of hypomethylated (41.21%) DMSs than hypermethylated (24.26%) in the intergenic region. We then determined the DMRs and found that the hypomethylated DMRs (*n* = 599) outnumbered the hypermethylated (*n* = 426) DMRs in CD4^+^ T cells of *Egr2^−/−^*B6/*lpr* compared with the controls (Fig. 4C). Also, we observed more hypomethylated DMRs in the *Dlk1*-*Dio3* locus in *Egr2^−/−^*B6/*lpr* mice (Table III). Our data revealed that *Egr2* deletion led to methylation loss at the global level and at the *Dlk1*-*Dio3* locus.

**Table III.** DMRs at *Dlk1-Dio3* region of *Egr2*^−/−^ B6/*lpr* compared with *Egr2^fl/fl^*B6/*lpr* mice

Chrom	Start	End	Length	nCG	Meth Diff	*Egr2*^−/−^B6/*lpr* Mean Meth	*Egr2*^fl/fl^B6/*lpr* Mean Meth
chr12	109560215	109560448	234	5	−0.10493	0.85655	0.96148
chr12	109828129	109828488	360	5	0.13927	0.74218	0.60291
chr12	109988263	109988389	127	5	−0.1327	0.76127	0.89397
chr12	110114252	110114532	281	5	−0.11043	0.67799	0.78842
chr12	110176792	110177223	432	11	−0.14076	0.55261	0.69337

Chrom, chromosome; Meth Diff, methylation difference; nCG, number of cytosine-guanine.

## Discussion

The dysregulation of miRNAs located at the genomic imprinting *Dlk1*-*Dio3* locus has been identified in various types of cancers and autoimmune diseases, including MS and lupus. Although direct evidence to show the relationship between *Dlk1*-*Dio3* miRNAs and lupus pathogenesis is still lacking, Vinuesa et al. (48) have reported that 49 of 72 autoimmune genes were predicted to be the targets of *Dlk1*-*Dio3* miRNAs. The Kyoto Encyclopedia of Genes and Genomes pathway enrichment analysis of the predicted target genes of the *Dlk1*-*Dio3* miRNAs that are upregulated in murine lupus or in MS showed the enrichment of signaling cascades that have been implicated in autoimmune disease pathogenesis (Supplemental Fig. 1) (20, 21). For example, the *Dlk1*-*Dio3* miRNAs targeted the PI3K/Akt/mTOR signaling pathway, which has been associated with the pathogenesis of lupus nephritis and MS/experimental autoimmune encephalomyelitis (49, 50). In addition, there is a significant enrichment of Ras-MAPK signaling, FcγR-mediated phagocytosis pathway, TCR signaling, cAMP signaling pathway, HIF-1 signaling, and Wnt signaling pathways in the predicted target genes of lupus-related *Dlk1*-*Dio3* miRNAs, which are also critically involved in autoimmunity and lupus pathogenesis (51–55) (Supplemental Fig. 1). In this study, we reported that *Egr2* deletion significantly reduced maternally expressed *Dlk1*-*Dio3* miRNAs, suggesting that EGR2 plays an important role in the upregulation of *Dlk1*-*Dio3* miRNAs in murine lupus CD4^+^ T cells. These data provide us with new insight into the autoimmunity regulatory role of EGR2 and new knowledge in understanding the molecular regulation of *Dlk1*-*Dio3* miRNAs in lupus.

The expression of the genes at the genomic imprinted *Dlk1*-*Dio3* locus was controlled by the two major differentially methylated regions, IG-DMR and Gtl2-DMR (MEG3-DMR in humans), and other methylation changes across the *Dlk1*-*Dio3* locus (56, 57). Our previous report showed the correlation between the upregulation and *Dlk1*-*Dio3* miRNAs and global DNA hypomethylation in MRL/*lpr* CD4^+^ T cells (19). Our RRBS analysis consistently revealed a DNA methylation loss at the whole-genome level and also at different genomic regions of MRL/*lpr* CD4^+^ T cells when compared with control MRL CD4^+^ T cells. Importantly, we identified three hypomethylated DMRs at the *Dlk1*-*Dio3* locus of MRL/*lpr* CD4^+^ T. These data suggested a link between the upregulation of *Dlk1*-*Dio3* miRNAs and site-specific DNA demethylation at the *Dlk1*-*Dio3* region. Additional study is necessary to determine whether these three hypomethylated DMRs contribute directly to the upregulation of *Dlk1*-*Dio3* miRNAs in MRL/*lpr* CD4^+^ T cells.

Reduced *Dnmt1* and/or *Dnmt3a/b* expression has been correlated with the global DNA hypomethylation in the human lupus (40, 41). However, unchanged *Dnmt1* expression and even increased *Dnmt3a/b* gene expression have also been identified in human lupus PBMCs or CD4^+^ T cells in different studies, despite the consistency of DNA hypomethylation in these lupus cells (58–60). An early study has shown that MRL/*lpr* CD4^+^T cells have reduced *Dnmt1* mRNA levels, correlating with global DNA hypomethylation and disease development in MRL/*lpr* mice (61). It is noteworthy that in this study, the authors compared the expression of *Dnmt1* in CD4^+^ T cells from MRL/*lpr* mice with active disease (16 wk of age) with CD4^+^ T cells from MRL/*lpr* at the prediseased stage (5 wk of age) but not with age-matched MRL control mice (61). In this article, we reported an increase of *Dnmt1* and *Dnmt3b* in CD4^+^ T cells of MRL/*lpr* mice when compared with age-matched MRL controls. With the finding of an increased expression of *Tet1* and *Tet2* in MRL/*lpr* CD4^+^ T, we speculate that the TET-mediated active DNA demethylation pathway likely plays a major role in the global DNA hypomethylation in CD4^+^ T cells of MRL/*lpr* mice. However, we cannot rule out the involvement of DNMT-mediated passive DNA demethylation pathway, because *Dnmt* gene expression level may not reflect the protein expression level and/or activity. Although *Dnmt1*, *Dnmt3b*, *Tet1*, and* Tet3* were significantly increased in CD4^+^ T cells of MRL*/lpr* mice, they were either reduced (*Dnmt3b and Tet1)* or not changed (*Dnmt1* and *Tet3*) in whole splenocytes of MRL/*lpr* mice when compared with MRL controls (Supplemental Fig. 2). This suggested that either DNMT- or TET-mediated DNA methylation changes could be cell- and tissue-specific and/or signal pathway-dependent (13).

EGR2 was initially identified as a vital regulator of the development of the hindbrain (62). The critical and complex roles of EGR2 in immunity and autoimmunity have now been extensively studied (25–32, 63–65). We recently reported that the EGR2 apparently had a differential immune regulatory role in normal B6 mice *versus* autoimmune-prone B6/*lpr* mice (31). In contrast to how conditional *Egr2* deletion in lymphocytes of normal B6 mice promoted autoantibody production and induced lupus-like disease, conditional *Egr2* deletion in B6/*lpr* mice had the opposite effect, with reduced autoantibodies, Ab production, and pathogenic double-negative T cells (31). In the current study, we found that *Egr2* deletion in B6/*lpr* mice significantly reduced the expression of DNA methylation–sensitive *Dlk1*-*Dio3* miRNAs, which are upregulated and implicated in the pathogenesis of autoimmune diseases such as lupus and MS (3, 19, 36, 48). It is possible that EGR2 regulates autoimmunity in lupus mice via the regulation of *Dlk1*-*Dio3* miRNAs.

To further understand how EGR2 regulates the expression of methylation-sensitive *Dlk1*-*Dio3* miRNAs, we investigated *Egr2* deletion–induced methylation changes in CD4^+^ T cells of B6/*lpr* mice. EGR2 has recently been shown to interact with TET2 to induce site-specific active DNA demethylation during monocyte differentiation, suggesting an epigenetic regulatory role of EGR2 in immune cells (47). We therefore initially thought that *Egr2* deletion might increase DNA methylation at *Dlk1*-*Dio3* locus to suppress *Dlk1*-*Dio3* miRNAs expression. To our surprise, *Egr2* deletion induced a greater loss of DNA methylation than gain of DNA methylation in CD4^+^ T cells of *Egr2^−/−^*B6/*lpr* mice at whole genome level (Fig. 4) and at the *Dlk1*-*Dio3* locus (Table III). Researchers have devoted considerable attention to the reverse relationship between gene expression level and DNA methylation level at the genome, particularly in the promoter region. Nevertheless, the role of DNA methylation in gene expression is much more intricate and depends on the methylation patterns at genomic sites, cell and tissue types, and developmental stages (66–68). For example, in both plant and human systems, a positive correlation between the DNA methylation within the intragenic regions (gene body) and gene expression was observed (68, 69). Further, DNA hypermethylation has been associated with the upregulation of gene expression in prostate cancer tissues (70). Thus, although there is a correlation between the expression of *Dlk1*-*Dio3* miRNAs and global DNA hypomethylation, as well as site-specific DNA hypomethylation, in MRL/*lpr* CD4^+^ T cells, we cannot exclude the possibility that *Egr2* deletion–induced hypomethylated DMRs at *Dlk1*-*Dio3* locus might contribute to the suppression of *Dlk1*-*Dio3* miRNAs in *Egr2*^−/−^B6/*lpr* mice. It is worth noting that the *Egr2* deletion–induced DMRs in B6/*lpr* mice do not overlap with the hypomethylated DMRs that were determined in MRL/*lpr* mice. A site-specific modulation of the methylation status at *Egr2* deletion–induced DMRs may provide insights into the direct role of EGR2 in the epigenetic regulation of *Dlk1*-*Dio3* miRNAs. Additionally, as a transcription factor, EGR2 may regulate the expression of *Dlk1*-*Dio3* miRNAs without altering the methylation.

The current understanding of the role of EGR2 in immunity and autoimmunity is primarily based on data derived from genetically modified B6 mice that may not fully capture the function of EGR2 in diverse pathological contexts. Our study revealed that *Egr2* deletion in autoimmune-prone B6/*lpr* mice altered the methylation at the genome-wide level and suppressed the expression of methylation-sensitive *Dlk1*-*Dio3* miRNAs. These data provide us with a fresh perspective on comprehending the pathogenic contribution of upregulated EGR2 in lupus, although the detailed mechanism of EGR2 regulation of *Dlk1*-*Dio3* miRNAs needs to be further investigated.

## Supplementary Material

Supplemental Figures 1 (PDF)
