# Supplementary material for: Egr2 Deletion in Autoimmune-Prone C57BL6/lpr Mice Suppresses the Expression of Methylation-Sensitive Dlk1-Dio3 Cluster MicroRNAs
Source: Immunohorizons. 2023 Dec 28;7(12):898–907. doi: 10.4049/immunohorizons.2300111 (PMC10759154; doi:10.4049/immunohorizons.2300111)
Supplement: Supplemental Figures 1 (PDF) [file IH_2300111_Supplemental_1.pdf]

Supplemental Data

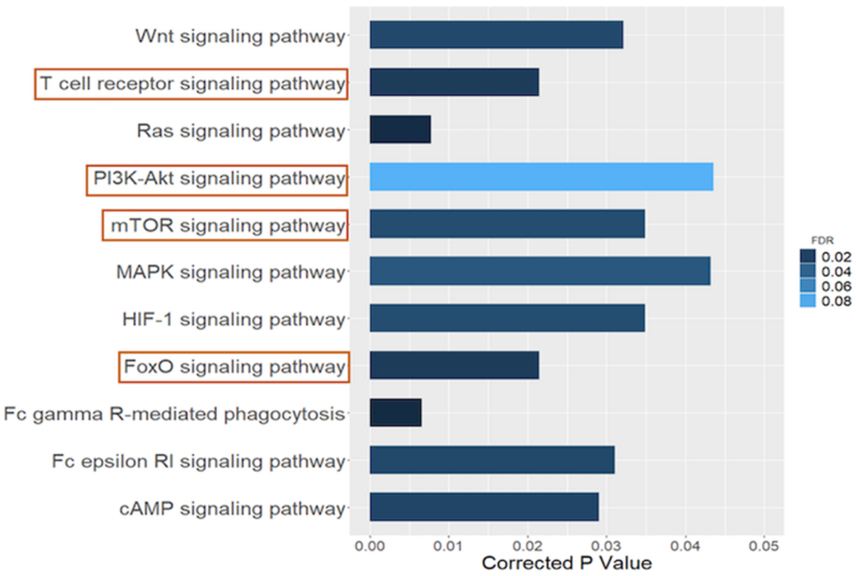

Supplemental Figure 1. Significant enrichment of autoimmune lupus-related pathways that are predicted to be targeted by the upregulated *Dlk1-Dio3* miRNAs. The target genes of miR-127, miR-154, miR-299, miR-300, miR-329, miR-376b, miR-379, miR-382, miR-411, miR-433, and miR-494 were predicted by miRDB (http://mirdb.org). KEGG (Kyoto Encyclopedia of Genes and Genomes) pathway enrichment analysis was performed in R. B-H procedure was performed to calculate the corrected *p*-value and the false discovery rate (FDR). The cutoff corrected *p*-value was set to 0.05 and the cutoff FDR was set to 0.1 for screening the candidate pathways that are significantly enriched in the target genes.

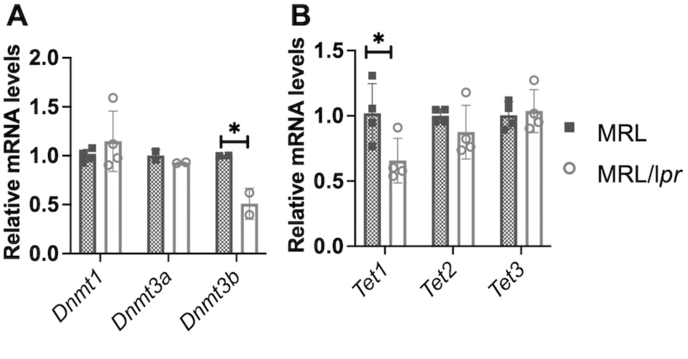

Supplemental Figure 2. Reduced *Dnmt3b* and *Tet1* gene expression in the splenocytes of MRL/*lpr* mice compared to control MRL mice. RT-qPCR was performed to quantify the expression levels of *Dnmt* and *Tet* genes in splenocytes from female MRL/*lpr* (14-15 weeks old) and sex- and age-matched control MRL mice. **(A)** The graph showed a reduction of *Dnmt3b* and no change in the expression of *Dnmt1/3a* in MRL/*lpr* splenocytes. **(B)** Reduction of *Tet1* and no changes in the expression of *Tet2/3* in MRL/*lpr* splenocyte*s*. Horizontal bars in the summary graphs indicated mean ± SD (n≥2). Unpaired student *t* tests were performed; *, p<0.05.
